# Acute Human Self-Poisoning with Imidacloprid Compound: A Neonicotinoid Insecticide

**DOI:** 10.1371/journal.pone.0005127

**Published:** 2009-04-08

**Authors:** Fahim Mohamed, Indika Gawarammana, Thomas A. Robertson, Michael S. Roberts, Chathura Palangasinghe, Shukry Zawahir, Shaluka Jayamanne, Jaganathan Kandasamy, Michael Eddleston, Nick A. Buckley, Andrew H. Dawson, Darren M. Roberts

**Affiliations:** 1 South Asian Clinical Toxicology Research Collaboration, Department of Clinical Medicine, University of Peradeniya, Peradeniya, Sri Lanka; 2 Therapeutics Research Unit, School of Medicine, University of Queensland, Brisbane, Australia; 3 Polonnaruwa General Hospital, North Central Province, Polonnaruwa, Sri Lanka; 4 Anuradhapura General Hospital, North Central Province, Anuradhapura, Sri Lanka; 5 Scottish Poisons Information Bureau, Royal Infirmary of Edinburgh, and Clinical Pharmacology Unit, University of Edinburgh, Edinburgh, United Kingdom; 6 Medical Professorial Unit, POW Hospital Clinical School, University of New South Wales, Kensington, Australia; 7 School of Medicine and Public Health, University of Newcastle, Callaghan, Australia; 8 Burns, Trauma and Critical Care Research Centre, University of Queensland, Brisbane, Australia; University of Swansea, United Kingdom

## Abstract

**Background:**

Deliberate self-poisoning with older pesticides such as organophosphorus compounds are commonly fatal and a serious public health problem in the developing world. The clinical consequences of self-poisoning with newer pesticides are not well described. Such information may help to improve clinical management and inform pesticide regulators of their relative toxicity. This study reports the clinical outcomes and toxicokinetics of the neonicotinoid insecticide imidacloprid following acute self-poisoning in humans.

**Methodology/Principal Findings:**

Demographic and clinical data were prospectively recorded in patients with imidacloprid exposure in three hospitals in Sri Lanka. Blood samples were collected when possible for quantification of imidacloprid concentration. There were 68 patients (61 self-ingestions and 7 dermal exposures) with exposure to imidacloprid. Of the self-poisoning patients, the median time to presentation was 4 hours (IQR 2.3–6.0) and median amount ingested was 15 mL (IQR 10–50 mL). Most patients only developed mild symptoms such as nausea, vomiting, headache and diarrhoea. One patient developed respiratory failure needing mechanical ventilation while another was admitted to intensive care due to prolonged sedation. There were no deaths. Median admission imidacloprid concentration was 10.58 ng/L; IQR: 3.84–15.58 ng/L, Range: 0.02–51.25 ng/L. Changes in the concentration of imidacloprid in serial blood samples were consistent with prolonged absorption and/or saturable elimination.

**Conclusions:**

Imidacloprid generally demonstrates low human lethality even in large ingestions. Respiratory failure and reduced level of consciousness were the most serious complications, but these were uncommon. Substitution of imidacloprid for organophosphorus compounds in areas where the incidence of self-poisoning is high may help reduce deaths from self-poisoning.

## Introduction

Intentional self-poisoning with pesticides is an important public health problem in the Asia- Pacific region with an estimated 300,000 deaths occurring each year [1], [2]. A large number of these deaths are due to poisoning with organophosphorus insecticides which are an integral part of agriculture within this region [2]. Due to the intrinsic toxicity of these compounds, new pesticides continue to be developed and released to the market which almost always occurs in the absence of data on direct human toxicity. Instead, human toxicity is often extrapolated from toxicological studies in animals, the relevance of which is poorly defined. Therefore, data reporting the outcomes from human exposures to these newer insecticides are required. This information can assist in the risk assessment and clinical management of patients with acute exposures and support policy decisions by regulatory agencies. Previous restrictions in the availability of highly toxic compounds appeared to substantially reduce deaths from poisoning [3], [4], [5] without harming agricultural outputs [5].

The neonicotinoids are a new major class of highly potent insecticides that are used for crop protection and flea control [6]. Insecticides within this class include imidacloprid, acetamiprid, clothianidine, and thiocloprid. These insecticides are agonists at the nicotinic acetylcholine receptors (nAChRs), particularly the α4β2 subtype [7], [8], which induces neuromuscular paralysis and eventually death. They are highly selective for nAChRs in insects compared with mammals, which should reduce morbidity and mortality in cases of human poisoning [7]. If clinical data on human exposures support this, they may potentially replace the more widely used cholinesterase inhibitors (organophosphorus and carbamate compounds) in crop protection.

**Figure 1:**
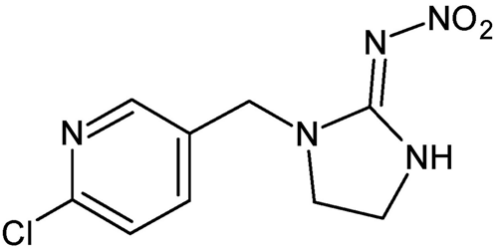
Chemical structure of imidacloprid.

Imidacloprid (CAS 138261-41-3; figure 1) is the most commonly used neonicotinoid insecticide in Sri Lanka. On the basis of animal studies it is classified as moderately hazardous (Class II WHO; toxicity category II EPA) [9], [10]. It has low acute lethal toxicity to mammals, birds, and fish: the acute oral LD_50_ (dose that is lethal in 50% of animals) of imidacloprid in rats is 475 mg/kg and the acute dermal LD_50_ exceeds 5000 mg/kg. It also does not cause eye irritation (rabbits) or skin sensitization (guinea pigs) [11].

Data on human exposure to imidacloprid is limited to occupational exposures [12], [13], [14], [15] and 13 case reports of self-poisoning [14], [16], [17], [18], [19], [20], [21]. Mild clinical effects such as tachycardia, hypertension, mydriasis, nausea and vomiting occur, but more serious sequelae including respiratory failure, seizures [15], [17], [20] and even death [16], [18], [21] are reported. This raises serious doubts about its assumed superior safety profile over older insecticides. However, in the majority of cases the concentration of imidacloprid was not quantified so it was not possible to confirm exposure or consider dose in the risk assessment.

In this study we sought to further describe the spectrum of toxicity and clinical outcomes relative to the admission plasma concentration in patients with acute imidacloprid poisoning.

## Methods

### Ethics statement

This observational study was approved by Human Research Ethics committees of the University of Colombo Faculty of Medicine, The Sri Lankan Medical Association, The Australian National University and Oxfordshire, UK. Ethics approval includes provision of a single blood sample on admission, regular clinical reviews during hospitalisation and publication of de-indentified clinical data. Multiple blood samples were obtained as part of a smaller sub-study which required additional written consent. In all cases, informed verbal consent was obtained from the patient or a relative in their native language.

### Set up

A prospective observational cohort study of all poisoning presentations was established during 2002 in three hospitals in the North Central and North Western provinces of Sri Lanka and the cohort was extended to Central province during the year 2005.

### Inclusion and exclusion criteria

All patients presenting to a study hospital with a history of imidacloprid exposure were considered for this study. Patients under 14 years, pregnant women and patients presenting with co-ingestions are excluded from the study.

### Data collection procedure

Clinical observations of all patients with imidacloprid poisoning were prospectively recorded on a specially designed data base from March 2002 to March 2007. The poison ingested was identified from the patient's or relative's history, examination of the bottle label and/or the doctor's comments on transfer letters. Blood tests such as full blood count, biochemistry or cholinesterase activity were not performed prospectively as there is limited availability of such services in these predominantly rural hospitals.

Blood samples were collected from patients for quantification of the concentration of imidacloprid and other biochemical assays at a later date. Following collection, the plasma was promptly separated and samples were stored at −23°C and transported to the University of Queensland, Australia on dry ice for analysis. Samples were analyzed by HPLC (Shimadzu) with MSMS detection (Applied Biosystems API2000) of imidacloprid (MRM 255.9/208.9) and internal standard d4-Imidacloprid (MRM 260.0/213.1) at 3.7 min. Separation of the imidacloprid peak was performed using Strata C18 (5 mm×2 mm) online solid phase extraction and Gemini (50 mm×2 mm) analytical columns (Phenomenex) using a standard valve configuration [22], the 3 minute equilibration step and 6 minute gradient shown in table 1. Solvent A (Pumps A & B) and Solvent B (Pump C) contained 0.1% formic acid with acetonitrile, water and methanol in the ratios 5∶95∶0 and 90∶5∶5, respectively. Samples were prepared by combining plasma (10 µL), d4-imidacloprid (2 µg/mL) in zinc (II) sulfate solution (100 mM, 20 µL) and acetonitrile (50 µL). Each sample was prepared by combining the three components, vortex mixing (5 sec), centrifugation (5 min at 4000 rpm), transfer of supernatant (∼70 µL) and LCMS injection of 50 µL (Table 1).

**Table 1:** LC Events

LC Events			
Time (min)	device	parameter	value
−3	Valve	Switch	A
−3–0	Pumps	Flow (mL/min)	0.8
		%A∶%B∶%C	50∶50∶0
0–1.45	Pumps	Flow (mL/min)	0.2
		%A∶%B∶%C	100∶0∶0
1.50	Valve	Switch	B
1.50		%A∶%B∶%C	0∶100∶0
1.50–6.00	Pumps	Flow (mL/min)	0.6
4.00	Pumps	%A∶%B∶%C	0∶0∶100
4.50	Pumps	%A∶%B∶%C	0∶100∶0
6.00	Controller	stop	

Biochemical analyses were conducted by Queensland Health Forensic and Scientific Services at Princess Alexandra Hospital, Australia. This service is accredited by the National Association of Testing Authorities, Australia and certified to International Standards (ISO 9001).

Data were entered in to an excel sheet and analyzed using the statistical Program STATA IC 10.

## Results

Over the 5 year period, 68 patients presented to study hospitals with a history of imidacloprid exposure. Seven cases were occupational dermal exposures, all of whom remained asymptomatic and were discharged within 24 hours of admission. Five patients reported co-ingestion with another pesticide and were excluded from further analysis, leaving 56 patients with acute imidacloprid self-poisoning.

The median time to present to a study hospital since ingestion was 4 hours (IQR 2.3–6.0 hours). The median volume reported as ingested in self-poisoning was 15 mL (IQR: 10–50); although in 23 the volume ingested was unknown.

The majority of patients (54/56) had only mild symptoms such as nausea, vomiting, headache, dizziness, abdominal pain, and diarrhoea during the hospital stay which was largely self-resolving. The median Glasgow Coma Score (GCS) on presentation was 15 (IQR: 10–15). There were no deaths giving a case fatality of 0% (95% CI: 0.0–5.2%). However, two patients developed more severe symptoms requiring management in an intensive care unit and are described in more detail below.

### Case 1

**Figure 2:**
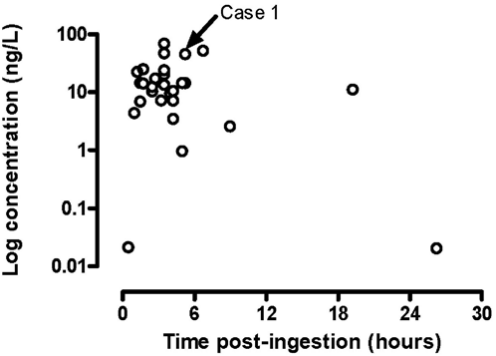
Admission imidacloprid plasma concentrations (n = 33). Compared to other cases of imidacloprid poisoning where the plasma concentration was quantified (12.5 and 2.05 ng/L post-mortem [16]), our patients survived despite relatively high concentrations.

A 35 year old woman was admitted to a peripheral hospital soon after ingestion of an unknown amount of imidacloprid. Due to an initial lack of history, she had been managed as a case of organophosphorus pesticide poisoning. She received forced emesis, 1.2 mg of atropine and 1g of pralidoxime before her transfer to the study hospital. At this time, 2 hours post ingestion, she was agitated and had a blood pressure of 110/70 mmHg, regular pulse rate of 120/minute, pupil diameter 3 mm bilaterally and clear lungs. She received a bolus dose of haloperidol (5 mg intramuscularly) for agitation. At 16 hours after ingestion she developed a respiratory arrest requiring endotracheal intubation using atracurium 25 mg and midazolam 5 mg. She received an atropine infusion at 1.2 mg/hour and prophylactic cefuroxime 750 mg 8 hourly and metronidazole 500 mg every 8 hours for suspected pulmonary aspiration. On her second day in ICU, she became hypotensive which was treated with dopamine infusion. Other treatments included regular pralidoxime (1g every 6 hours) and chest physiotherapy. She was extubated on her 4^th^ ICU day after 3 days of mechanical ventilation and discharged home 9 days post-ingestion with no apparent residual effects. During her recovery the patient reported a history of imidacloprid only. This was subsequently confirmed on laboratory testing of a blood sample obtained 5 hours post-ingestion when the plasma concentration of imidacloprid was 44.6 ng/L (Figure 2) and the butyryl cholinesterase activity was normal.

### Case 2

A 26 year old man presented to a peripheral hospital following ingestion of an unknown amount of imidacloprid under the influence of alcohol. He received forced emesis and atropine (3 mg bolus followed by infusion of 2 mg/hour) and was then transferred to one of the study hospitals. On admission to the study hospital (4.5 hours post-ingestion) he had vomiting, a regular pulse rate of 84/minute, blood pressure 100/80 mmHg, pupil diameter 6 mm bilaterally, respiratory rate 40/minute, pulse oximetry was 100% and GCS 3/15. The patient was transferred to the ICU 9 hours post-ingestion for closer monitoring. He received nebulised salbutamol and intravenous cefuroxime and metronidazole for prophylaxis against aspiration pneumonia. His clinical condition improved within 24 hours and he was discharged alive 3 days later. Blood samples were not available to confirm exposure in this patient.

### Toxicokinetics and biochemistry

Of the 56 patients with imidacloprid self-poisoning, 13 patients provided serial blood samples, 38 patients provided a single blood sample, and 5 patients refused to give any samples. Blood samples from the first 33 cases were analysed as described and the results of imidacloprid quantification are shown in figure 2. Exposure was confirmed in 28 patients, with a median admission plasma concentration of 10.58 ng/L; IQR: 3.84–15.58 ng/L and range: 0.02–51.25 ng/L. In 5 patients the concentration was less than the level of quantification (0.008 ng/L), consistent with minimal exposure to imidacloprid.

**Figure 3:**
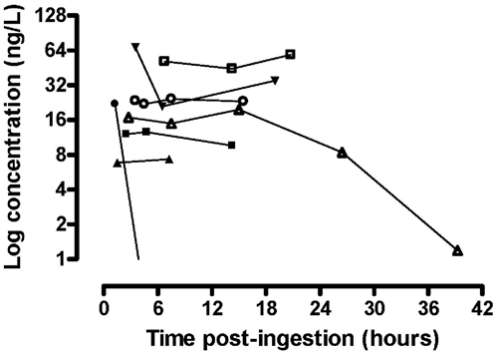
The toxicokinetics of imidacloprid in patients with self-poisoning (n = 8 patients). The concentration was high on admission and remained elevated in the majority of patients suggesting either prolonged absorption and/or elimination.

Imidacloprid was only detected in eight of the patients who provided serial blood samples but in one patient the plasma concentrations were all less than 0.3 ng/L. The concentration-time profiles for these seven patients are shown in Figure 3 and demonstrate a rapid initial absorption with high concentrations being noted on admission. However, the concentration remained elevated for up to 10–15 hours post-ingestion, which might suggest that absorption and/or elimination are saturable (zero-order) or prolonged at high doses. In one of the patients there was a rapid decrease in concentration soon after the admission sample which might represent a distribution phase; the reason for this sample differing from the others is not apparent from this data. Quantification of metabolite production may further define the toxicokinetics underpinning these observations, but unfortunately these were not able to be conducted at this time.

### Biochemistry

Admission blood samples from the same 33 patients were screened for biochemical abnormalities. No major abnormalities were noted in terms of electrolytes, blood glucose, renal function and liver function tests. Minor abnormalities included median venous bicarbonate of 14 mmol/L (IQR 10–15 mmol/L) and the median anion gap was raised at 20 mmol/L (IQR 18–26 mmol/L). We were not able to perform arterial blood gases which might have confirmed the presence of high anion gap metabolic acidosis. Median creatine kinase (CK) was measured at 115 IU/L (IQR 75–124), which is within the commonly quoted reference range and troponin-I was not elevated.

## Discussion

This is the only prospective human case series reporting outcomes from acute self-poisoning with the neonicotinoid insecticide imidacloprid. We demonstrated that imidacloprid self-poisoning resulted in mostly minor toxicity with a case-fatality of 0%. This is favourable compared to outcomes with other insecticides, in particular the widely used organophosphorus compounds which commonly have a case fatality between 5 and 30% [23], [24]. The most severely poisoned patients were both administered antidotes used for the treatment of organophosphorus pesticides (a common cause of poisonings in the region) and this may have increased the apparent toxicity. Many patients may have had a moderate metabolic acidosis on admission; however, simple supportive care was sufficient to ensure a good outcome for all patients in this series.

Tachycardia and hypertension have usually been reported in previous cases, and recurrent ventricular fibrillation was the reported cause of death in a 69 year-old woman with coronary artery disease [18]. Only 2 patients in our case series developed any cardiovascular toxicity which was predominantly hypotension and biomarkers of cardiac toxicity were not elevated. While electrocardiographic monitoring was not conducted in these patients, blood pressure improved with intravenous fluids. Therefore serious arrhythmias were unlikely to have caused the hypotension.

Biochemical abnormalities and rhabdomyolysis have been reported as potentially serious complications that might lead to mortality [15], [21]. Most of the patients in our series had normal CK and biochemistry with the exception of low venous bicarbonate. The cause of this is not clear given the other biochemical results, although diarrhoea may be contributory. It may also be due to acidic metabolites of imidacloprid such as 6-chloronicotinic acid and other metabolites [6]; however, metabolic pathways of imidacloprid have not been extensively studied in humans. Direct mitochondrial toxicity from a component of the formulation (as observed with some other pesticides) may cause anaerobic metabolism and produce a lactic acidosis which may cause a moderate decrease in bicarbonate.

In animals, imidacloprid penetrates the blood-brain barrier to only a very limited extent [7]. While a decreased level of consciousness was uncommon in our study, prolonged sedation and respiratory depression was noted in two patients which may have been due to co-ingestion of ethanol. Transient respiratory impairment appeared to contribute to deaths reported in patients with severe poisoning where co-ingestion of ethanol was not reported [20], [21].

There are no specific antidotes for neonicotinoid poisoning in mammals [7], [25]. On the basis of our experience, symptomatic and supportive care is all that is required for the management of patients with acute imidacloprid poisoning. Treatment with oximes such as pralidoxime is expected to be either ineffective or contraindicated. Oximes in the absence of organophosphorus pesticides have a weak inhibitory effect on acetylcholinesterase activity and therefore might increase nicotinic effects (tachycardia, hypertension, muscle weakness). It is notable that our two most seriously poisoned cases received treatment with pralidoxime.

The concentration-time profile shown in Figure 3 suggests that there is rapid absorption, with high concentrations being noted on admission. In rats, imidacloprid is rapidly and almost completely absorbed (>92%) from the gastrointestinal tract. The peak plasma concentration is observed within approximately 2.5 hours and is followed by a rapid disposition phase. However, in our patients the concentrations generally remained elevated for up to 10–15 hours post-ingestion, which might suggest saturation of one or more kinetic (absorption or elimination) pathways in humans at high doses. A possible factor influencing the observed kinetic profile is the administration of atropine (commonly given routinely to insecticide poisonings in Sri Lanka) which is known to prolong the absorption phase of xenobiotics [26].

Of the patients who provided serial samples, the final blood sample was generally obtained from patients around the time of discharge, when they appeared to be in good health. It is noted in figure 3 that for many of these patients the imidacloprid concentration remained elevated. Therefore, plasma concentrations do not appear to be useful for guiding clinical management, which may reflect the contribution of metabolites or co-formulants. In rats, the metabolism of imidacloprid is rapid and extensive where only 10–16% of a dose is excreted unchanged [6]. Metabolites may contribute to human toxicity as they do in insects, in particular the olefin metabolite which retains insecticidal activity and nAChR activity [7]. Potentially, individual variation in cytochrome P450 isoenzymes involved in oxidative imidacloprid metabolism may contribute to variable toxicity [7], [27]. Admire SL 200® (200 g/L), the most popular imidacloprid-containing product in Sri Lanka, contains dimethylsulfoxide and N-methylpyrolidone as solvents which are irritants and may induce gastrointestinal toxicity.

Four deaths have been reported in the literature, and the post-mortem blood concentrations in two cases were 12.5 and 2.05 ng/L [16], which surprisingly is not substantially greater than the median plasma concentration in our study (9.86 ng/L). However, ante-mortem plasma concentrations were not reported in these two fatalities and a direct comparison of the concentrations may be misleading. There are no data on the blood/plasma concentration ratio or post-mortem redistribution.

This study demonstrates that an acute ingestion of 20% SL formulations of imidacloprid, even following large ingestions in patients with self-poisoning, is relatively safe. Therefore, it may be advantageous to promote the use of imidacloprid or similar pesticides in areas where the incidence of self-poisoning is high. However, before this occur the relative risks and benefits of this insecticide (which has been debated)[28], [29], [30] must be compared to those of existing pesticides. This will require careful consideration by independent regulatory authorities.

Imidacloprid pesticides appear to be of low toxicity to humans causing only mild symptoms such as vomiting, abdominal pain, headache and diarrhoea in the majority of cases. Large ingestions may lead to sedation and respiratory arrest. Patients with a low GCS should be closely monitored for onset of respiratory compromise but most patients only need symptomatic and supportive care. More research is required to show if the replacement in agriculture of older anti-cholinesterase pesticides with newer pesticides with much lower in-hospital case-fatality will lead to an overall reduction in deaths from self-poisoning.

## References

[1] Jeyaratnam J (1990). Acute pesticide poisoning: a major global health problem.. World Health Stat Q.

[2] Eddleston M, Phillips MR (2004). Self poisoning with pesticides.. BMJ.

[3] Roberts DM, Karunarathna A, Buckley NA, Manuweera G, Sheriff MH (2003). Influence of pesticide regulation on acute poisoning deaths in Sri Lanka.. Bull World Health Organ.

[4] Gunnell D, Fernando R, Hewagama M, Priyangika WD, Konradsen F (2007). The impact of pesticide regulations on suicide in Sri Lanka.. Int J Epidemiol.

[5] Manuweera G, Eddleston M, Egodage S, Buckley NA (2008). Do targeted bans of insecticides to prevent deaths from self-poisoning result in reduced agricultural output?. Environ Health Perspect.

[6] JMPR (2001). Pesticide residues in food – 2000. Report of the Joint Meeting of the FAO Panel of Experts on Pesticide Residues in Food and the Environment and the WHO Core Assessment Group.. FAO Plant Production and Protection Paper 167.

[7] Tomizawa M, Casida JE (2005). Neonicotinoid insecticide toxicology: mechanisms of selective action.. Annu Rev Pharmacol Toxicol.

[8] Tomizawa M, Casida JE (2003). Selective toxicity of neonicotinoids attributable to specificity of insect and mammalian nicotinic receptors.. Annu Rev Entomol.

[9] Meister R (1995). 1995. Farm chemical handbook ’95. Meister Publishing Company.

[10] WHO (2005).

[11] Thyssen J, Machemer L (1999). Imidacloprid: Toxicology and metabolism..

[12] Faul J (1996).

[13] Calumpang SM, Medina MJ (1996). Applicator exposure to imidacloprid while spraying mangoes.. Bull Environ Contam Toxicol.

[14] Steefens W (2000).

[15] Agarwal R, Srinivas R (2007). Severe neuropsychiatric manifestations and rhabdomyolysis in a patient with imidacloprid poisoning.. Am J Emerg Med.

[16] Proenca P, Teixeira H, Castanheira F, Pinheiro J, Monsanto PV (2005). Two fatal intoxication cases with imidacloprid: LC/MS analysis.. Forensic Sci Int.

[17] Wu IW, Lin JL, Cheng ET (2001). Acute poisoning with the neonicotinoid insecticide imidacloprid in N-methyl pyrrolidone.. J Toxicol Clin Toxicol.

[18] Huang NC, Lin SL, Chou CH, Hung YM, Chung HM (2006). Fatal ventricular fibrillation in a patient with acute imidacloprid poisoning.. Am J Emerg Med.

[19] David D, George IA, Peter JV (2007). Toxicology of the newer neonicotinoid insecticides: imidacloprid poisoning in a human.. Clin Toxicol (Phila).

[20] Tamura M, Endo Y, Kuroki Y, Ohashi N, Yoshioka T (2002). [Investigation and case study of Imidacloprid insecticide caused poisoning].. Chudoku Kenkyu.

[21] Shadnia S, Moghaddam HH (2008). Fatal intoxication with imidacloprid insecticide.. Am J Emerg Med.

[22] Kahlich R, Gleiter CH, Laufer S, Kammerer B (2006). Quantitative determination of piritramide in human plasma and urine by off- and on-line solid-phase extraction liquid chromatography coupled to tandem mass spectrometry.. Rapid Commun Mass Spectrom.

[23] Rao C, Venkateswarlu V, Surender T, Eddleston M, Buckley NA (2005). Pesticide Poisoning in South India – Opportunities for Prevention and Improved Medical Management.. Trop Med Int Health.

[24] Eddleston M, Eyer P, Worek F, Mohamed F, Senarathna L (2005). Differences between organophosphorus insecticides in human self-poisoning: a prospective cohort study.. Lancet.

[25] Sheets LP (2002). The Neonicotinoid insecticides (2002)..

[26] Roberts DM, Buckley NA (2007). Pharmacokinetic considerations in clinical toxicology: clinical applications.. Clin Pharmacokinet.

[27] Schulz-Jander DA, Casida JE (2002). Imidacloprid insecticide metabolism: human cytochrome P450 isozymes differ in selectivity for imidazolidine oxidation versus nitroimine reduction.. Toxicol Lett.

[28] Bayer (2002). Imidacloprid-expert overview.. http://www.apiservices.com/articles/us/imidacloprid_bayer.htm.

[29] Bayer (2005). From Active substance to product-the formulation is key.. http://www.foodchainpartnership.bayercropscience.com/bayer/cropscience/cscms.nsf/id/For_Agro/$file/formulation.pdf.

[30] Cox C (2001). Imidacloprid insecticide factsheet.. Journal of pesticide reform.

